# Cow’s Milk Protein Allergy as a Model of Food Allergies

**DOI:** 10.3390/nu13051525

**Published:** 2021-04-30

**Authors:** Arianna Giannetti, Gaia Toschi Vespasiani, Giampaolo Ricci, Angela Miniaci, Emanuela di Palmo, Andrea Pession

**Affiliations:** 1 Pediatric Unit, IRCCS Azienda Ospedaliero-Universitaria di Bologna, 40138 Bologna, Italy; ariannagiannetti25@gmail.com (A.G.); angela.miniaci@aosp.bo.it (A.M.); emanuela.dipalmo@gmail.com (E.d.P.); 2 Specialty School of Paediatrics, Alma Mater Studiorum, University of Bologna, via G.Massarenti n 11, 40138 Bologna, Italy; gaia.toschi2@studio.unibo.it; 3 Department of Medical and Surgical Sciences (DIMEC), University of Bologna, via G. Massarenti n.9, 40138 Bologna, Italy; giampaolo.ricci@unibo.it

**Keywords:** cow’s milk allergy, oral food challenge, oral immunotherapy, diet, skin prick test, serum-specific IgE, breastfeeding

## Abstract

Cow’s milk allergy (CMA) is one of the most common food allergies in infants, and its prevalence has increased over recent years. In the present paper, we focus on CMA as a model of food allergies in children. Understanding the diagnostic features of CMA is essential in order to manage patients with this disorder, guide the use of an elimination diet, and find the best moment to start an oral food challenge (OFC) and liberalize the diet. To date, no shared tolerance markers for the diagnosis of food allergy have been identified, and OFC remains the gold standard. Recently, oral immunotherapy (OIT) has emerged as a new therapeutic strategy and has changed the natural history of CMA. Before this, patients had to strictly avoid the food allergen, resulting in a decline in quality of life and subsequent nutritional, social, and psychological impairments. Thanks to the introduction of OIT, the passive approach involving rigid exclusion has changed to a proactive one. Both the heterogeneity in the diagnostic process among the studies and the variability of OIT data limit the comprehension of the real epidemiology of CMA, and, consequentially, its natural history. Therefore, well-planned randomized controlled trials are needed to standardize CMA diagnosis, prevention, and treatment strategies.

## 1. Introduction

The prevalence of food allergies (FAs) and especially cow’s milk allergy (CMA, currently one of the most common FAs among children [1,2]) has increased in recent decades.

CMA is defined as a reproducible adverse reaction to one or more cow’s milk (CM) protein (usually casein or serum β-lactoglobulin) [3]. The underlying immunological mechanism, presentation times, and organs involved differentiate CMA from other adverse reactions to CM such as lactose intolerance [4].

**Figure 1:**
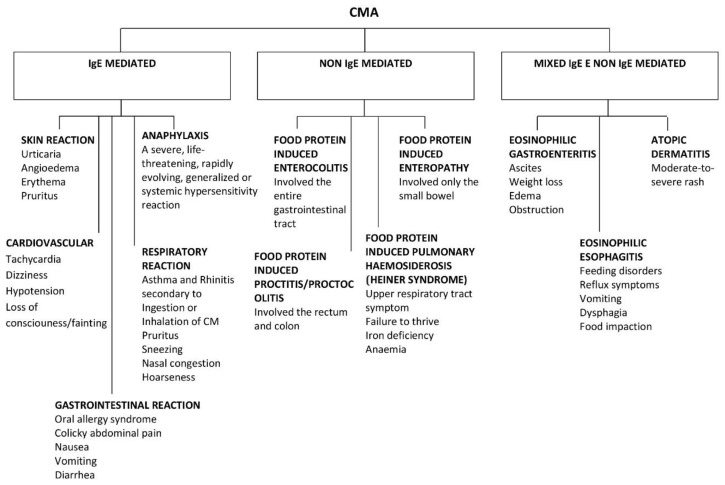
Clinical presentation of IgE and non IgE CMA.

CMA, like all FAs, can be divided into two main categories according to the type of immunological mechanism underlying it: immunoglobulin (Ig)E-mediated or non-IgE mediated [5] (Figure 1). IgE-mediated reactions are the most common. On the other hand, there are non-IgE mediated reactions that can arise from other cellular processes involving eosinophils or T-cells.

CMA usually occurs in the first 2 years of life and especially within the first year, unlike other allergies, such as peanut, tree nuts, fish and shellfish allergies which may develop later in childhood or adulthood. Most allergies (including CM allergies) resolve spontaneously during childhood or adolescence, whereas peanut and tree nut allergies are more likely to persist into adulthood [6,7].

## 2. Cow’s Milk Composition

CM contains from 30 to 35 g of protein per liter and many proteins, which are all potential allergens [8].

Through the acidification of raw skim milk to pH 4.6 at 20 °C it is possible to obtain two different fractions: the coagulum, containing the casein proteins which account for 80% of the fraction, and the lactoserum (whey proteins), representing 20% of the total milk proteins [9]. These proteins can also be divided into soluble and insoluble proteins [10].

The major milk allergens are soluble proteins, named whey proteins, which represent approximately 20% of total proteins [11]. Allergens present in the serum fraction include α-lactalbumin (Bos d 4) and β-lactoglobulin (Bos d 5), which are the most abundant, and immunoglobulins (Bos d 7), serum albumin (BSA, Bos d 6), and traces of lactoferrin, lysozyme, proteose-peptone, and transferrin [9]. In particular lactoferrin, lactoperoxidase, and lysozyme are important antimicrobial agents, while lactoferrin, β-lactoglobulin, and α-lactalbumin are important tumor suppressors [12].

The remaining 80% is represented by insoluble proteins known as caseins (known as Bos d 8). Total caseins can be divided into four proteins, representing different percentages of the whole fraction: αS1-casein, which is the most important (Bos d 9, 32%), as well as αS2-casein (Bos d 10, 10%), β-casein (Bos d 11, 28%), and κ-casein (Bos d 12, 10%). The main role of caseins relates to their mineral binding and carrier capacity, specifically for calcium and phosphorus [12].

CMAs are most frequently caused by whey proteins, but they can also be promoted by caseins [13]. As a matter of fact, most patients are sensitized to casein (Bos d 8), β-lactoglobulin (Bos d 5), and α-lactalbumin (Bos d 4), which are the major milk allergens. There are only a few studies that describe allergies to minor serum proteins such as immunoglobulin, bovine serum albumin, or lactoferrin [14].

CM contains at least 20 potentially allergenic proteins [15]. Most children with milk-allergy are sensitized to more than one allergen, with a greater variability of symptoms.

Table 1 provides a short synthesis of cow’s milk allergens and their characteristics.

## 3. Subtypes of Immune-Mediated Reactions to CM

CMAs have a range of clinical manifestations, with variable intensity. Moreover, clinical features can differ from “immediate” to “delayed” reactions, and this reflects the different pathogenesis (Figure 1).

Approximately 60% of CMA are IgE-mediated, although estimates change according to the study population and age [1].

The remaining 40% is divided into non IgE-mediated and mixed forms. The latter have different underlying mechanisms, presentations, and implications, which complicate the attempts to estimate the prevalence of CMA.

Non IgE-mediated CMAs are caused by less clear immune mechanisms. In this case, clinical findings are deferred and may occur 48 h or days after CM ingestion. Moreover, there are no specific symptoms or biomarkers that can guide the diagnosis, making it difficult to reach a conclusion. Typical non-IgE-mediated forms of CMA include CM enteropathy, food protein-induced proctitis/proctocolitis (FPIAP), food protein-induced enterocolitis syndrome (FPIES), and Heiner syndrome (pulmonary hemosiderosis) [1].

There are also mixed forms of CMA (both IgE- and non-IgE-mediated) that may have either humoral and/or cell-mediated mechanisms and may present with acute and/or chronic symptoms. These include atopic dermatitis, allergic eosinophilic esophagitis, and eosinophilic gastritis [1].

## 4. Prevalence of CMA

Before 1950, CMA was rarely diagnosed. Since 1970, significantly varying estimates of the incidence have been reported (ranging from 1.8% to 7.5%), reflecting the differences in diagnostic criteria and study design [16].

As a matter of fact, the prevalence estimates of CMA are affected by many factors. There is a marked heterogeneity in the prevalence of FA in the majority of papers. This could be the result of misleading differences in study design or methodology (including use of different definitions of CMA), or differences between populations and geographic areas [17].

Alongside the IgE, non-IgE, and mixed forms, there are also non-immune mediated reactions (i.e., intolerance), which are sometimes misclassified.

Another confounding factor while assessing prevalence is that many studies come from self-reports, with the consequent limitations linked to the subjective nature of the data [17]. Actually, the majority of studies on the epidemiology or natural history of FA have limitations. A precise evaluation requires a prospective ascertainment with a confirmatory oral food challenge (OFC) at predetermined intervals over time. For these reasons, studies such as these are rarely conducted due to their intrinsically reduced feasibility and ethical issues [17].

Therefore, determining an accurate diagnosis of CMA is fundamental. The process begins with an allergy-focused history that will guide all further investigations. In case that personal history is suggestive of allergy, a skin prick test (SPT) or specific IgE (sIgE) blood assay should be performed.

Evidence of sensitization (positive SPT or sIgE) with a suggestive history is usually sufficient to confirm the diagnosis, although OFC remains the gold standard [18].

It is important to emphasize that sensitization, i.e., raised sIgE directed against a specific antigen or positive SPT, in the absence of a supporting clinical history, is common in the general population but insufficient for a diagnosis of CMA. If diagnostic uncertainty remains even after a focused history and SPT/sIgE, an OFC is recommended to confirm diagnosis [18].

Other issues that may affect the estimates are the study design (prospective cohort vs cross-sectional), study population (demographic factors, geography, genetic/environmental factors), and natural history (incomplete identification of resolved cases) [1].

Despite these limitations during the assessment, a large number of studies in the United States and worldwide have attempted to estimate the prevalence of CMA [1].

An important contribution to prevalence studies was made by a meta-analysis performed by Rona et al., who analyzed publications from 1990 to 2005 and included only original studies (a total of 51 papers were considered appropriate for inclusion). The prevalence of self-reported FA was very high compared to that obtained using objective measures. Self-reported prevalence of CMA varied from 1.2% to 17%. The prevalence of CMA using SPT alone and sIgE alone, instead, was from 0.2% to 2.5% and from 2% to 9%, respectively. Studies using symptoms and sensitization (SPT ≥ 3 mm or sIgE > 0.35 kU/L) ranged from 0% to 2% prevalence, and those relying on OFC from 0% to 3% [19].

Another important meta-analysis and systematic review of CMA prevalence in Europe was performed by Nwaru et al., who analyzed publications from 2000 to 2012 (including 42 papers) [20]. The prevalence of self-reported CMA was 2.3% (95% CI 2.1–2.5), greater than that using SPT alone (0.3%, 95% CI 0.03–0.6) and sIgE alone (4.7%, 95% CI 4.2–5.1). The prevalence of CMA diagnosed by OFC was 0.6% (95% CI 0.5–0.8) and that using OFC or reported history of CMA was 1.6% (95% CI 1.2–1.9).

These meta-analyses show that prevalence estimates can be influenced by many factors such as geographic region, source population (high risk of referral bias vs general population), age and participation rates, and limitations of diagnosis [6].

Another important contribution was the EuroPrevall birth cohort study, published in 2015. In this study 12,049 children from nine different countries were enrolled, and 9336 (77.5%) were followed up until the age of 2 years. The authors calculated an overall incidence of challenge-proven CMA of 0.54% (95% CI 0.41–0.70) and showed differences in national incidences ranging from 1% (in the Netherlands and United Kingdom) to <0.3% (in Lithuania, Germany, and Greece). In this unique cohort study, they also showed that affected infants, without detectable specific antibodies to CM, were very likely to tolerate CM 1 year after diagnosis, whereas only half of those with specific antibodies in serum overcame their disease in the same period of time [21].

## 5. Diagnosis

Establishing an early and certain diagnosis of CMA is important to initiate the elimination diet in the case the diagnosis is confirmed or to avoid unnecessary dietary restrictions when it is not [22].

The diagnosis of CMA begins from the onset of signs and symptoms of CMA. Diagnosis is based on the combination of clinical history and physical examination, allergy tests such as sIgE and SPT, and, when indicated, OFC.

The first step is to evaluate the family and personal history in order to analyze every sign and symptom of the patient. Successively, it is fundamental to perform a differential diagnosis through laboratory evaluation and physical examination [23].

In addition to physical examination, there are objective assays used routinely in both epidemiological studies and clinical practice to investigate the condition, including sIgE and SPT. Despite the usefulness of sIgE antibodies (for tissue-bound and circulating IgE antibodies), these tests cannot differentiate between sensitization alone and clinical allergy [22]. The union of an evident history of allergic symptoms after CM exposure, associated with evidence of sensitization, certainly helps to make a definite diagnosis.

It has been demonstrated that the greater the food sIgE levels are and the SPT wheal size is, the higher the chances that the patient will manifest adverse reactions during an OFC. Numerous papers have analyzed the possibility of establishing a cutoff for sIgEs and SPTs for CM and its proteins that could predict whether a patient would react to an OFC [24]. Actually, several studies showed that cutoffs can vary with age [25], and many researchers are attempting to recommend diagnostic cutoffs for children [26,27]. However, cutoffs may change among different studies because of the type of allergen used to perform SPTs (commercial extract vs raw milk) or because of the degree of cooking [24].

Methods using sIgE measured [28] with in vitro immunoassays are still commonly referred to as IgE radioallergosorbent tests (RAST), and identify the level of IgE binding to specific proteins. Many studies have proposed a range of predictive cutoff values for the diagnosis of CMA, demonstrating a lack of agreement among different centers (Table 2).

Predictive cutoff values are found to be lower in younger children and increase with age [32], with diagnostic cutoff values remaining valid independently of the total serum IgE [37].

Therefore, it is difficult to assess standardized cutoffs for CM sIgE above which an OFC would not be required. Each patient would consequently need to be evaluated individually.

Traditionally sensitization is defined as the observation of a detectable sIgE level, (often sIgE > 0.35 kU/L but sometimes >0.10 kU/L) [1], although an OFC would be required if the sIgE level is positive but low.

SPTs have been used for decades to demonstrate or to exclude sensitization to allergens, as they are easy to perform, inexpensive, well tolerated, and provide immediately available results [22]. Traditionally, measured sensitization is often defined as a wheal at least 3 mm larger than the negative control [1]. Therefore, many studies aimed to avoid OFC by finding a cutoff of SPT able to predict a positive outcome of OFC [38] (Table 3).

A recent review by Cuomo et al. [24] reported that none of the cutoffs proposed in the literature could be used to definitively diagnose CMA. However, they found that in children aged <2 years, CMA diagnosis seemed to be highly likely when the sIgE reaction to CM extract was ≥5 kU/L, or when SPTs reactions with a commercial extract were above 6 mm, or when prick-by-prick reactions with fresh CM were above 8 mm [24].

However, if the clinical history is uncertain, SPT wheals measuring between 3 and 5 mm may be clinically irrelevant, and low levels of sIgE may be found in children without clinical CMA [33].

The negative predictive value of SPTs and sIgE is excellent (>95%) for immediate reactions [33]. Therefore, despite negative IgE tests, if there is a strong suspicion of CMA, an OFC is necessary to confirm the absence of a clinical allergy [13].

Despite this, even if OFC still remains the gold standard for CMA diagnosis (particularly the DBPCFC) [1], it is rarely required in clinical practice.

The OFC in CMA is performed by using baked or fresh milk. As baked milk is less allergenic in a context where a positive challenge is unexpected, it may be used initially because reactions are less likely to be severe [22].

OFCs should be performed under medical supervision in a hospital setting with an emergency kit available, especially in case of positive SPT or sIgE to CM and in infants at risk of an immediate reaction [13].

There is a lot of interest among clinicians in identifying markers that can predict the chance of developing tolerance and hence overcoming the allergy. Many studies have showed that IgE levels, expressed either as SPT wheal size or serum sIgE level, could be useful in discriminating between children who remained hypersensitive and those who became tolerant [44,45].The aim of most of these studies was to determine whether the monitoring of food sIgE levels over time could aid in the prediction of when patients would develop clinical tolerance. The likelihood estimates founded in these studies could help clinicians in providing prognostic information and in timing subsequent food challenges, thereby decreasing the number of premature and unnecessary DBPCFC [46].

Even if there is a lack of studies in this regard, some markers that may predict a persistent CMA have been identified over time. This explains the great interest of clinicians in finding a marker.

As a general rule, higher maximum IgE levels are associated with a reduced likelihood of developing tolerance [47].

Expressly regarding CMA, the association between increasing levels and persistence of allergy has been demonstrated, whereas decreasing levels indicate a faster recovery [48]. Low levels of IgG4 to β-lactoglobulin, instead, were found in children who required a longer elimination diet [36].

Vanto et al. [44] found that SPT wheal size <5 mm at diagnosis could be used to correctly identify 83% of individuals who developed tolerance at 4 years, whilst a wheal size ≥5 mm correctly identified 74% with persistent CMA.

These cutoff levels vary from study to study, possibly because of differences between the studied groups.

Garcia-Ara et al. [32] showed that sIgE levels predictive of clinical reactivity increased with age.

Shek and colleagues reported that the rate of reduction in food sIgE levels over time was predictive of the likelihood of developing tolerance in milk allergy. They were able to elaborate estimates of developing tolerance based on the reduction in sIgE levels, with a probability of tolerance of 31% in the case of a decrease by 50% in IgE levels, a probability of 45% in the case of a decrease by 70%, a probability of 66% when the decrease was 90%, and a probability of 94% for a decrease by 95% [46].

The value of sIgE has been analyzed with the aim of providing individuate cutoff levels related to the development of tolerance. Sampson et al. reported a positive predictive value of 95% in children with a median age of 3.8 years and CM-sIgE ≥ 15 kU/L [31]. Yavuz et al. examined sIgE levels which could predict a negative OFC in infants at different ages: by sIgE < 2.8 kU/L for children under 1 year, by 11.1 kU/L for children aged <2 years, and by <13.7 KU/l for children aged <6 years [49].

## 6. Risk Factors for CMA

The development of all FAs is influenced by genetics, environment, and genome–environment interactions, including epigenetic effects. Numerous risk factors for CMA have been identified or proposed that can contribute to allergy or sensitization.

There are unchangeable risk factors associated with a higher risk of FA, such as sex (male sex in children), race/ethnicity (increased among Asian and black children compared to white children), and family history of atopy [6]. It is generally presumed that atopy in parents increases atopic risk for the developing infant. This latter is one of the strongest risk factors, as occurs in other atopic diseases.

Koplin et al. discovered in a population of one-year-old infants with FA that the risk of FA increased to 40% in patients with one immediate family member with any allergic disease and to 80% in patients with two immediate family members with any allergic disease as compared to children no family history of allergy [50].

Controversy still exists as to whether a family history of atopy is also associated with a higher risk of CMA in infants. Goldberg et al., in a population-based study in 2013 [51], compared the parental atopic status of children with IgE-CMA (n = 66) with a group of healthy infants (n = 156). They reported no significant differences between the two groups and concluded that parental history of atopy alone cannot be used to anticipate which infants are at greater risk of developing IgE-mediated CMA [51].

On the contrary, in a recent paper by Sardecka et al. [52] on 138 infants with CMA and 101 healthy infants without allergy (with CMA confirmed by an elimination test and OFC), it was reported that the incidence of CMA was three times higher in infants with a positive family history for allergy. In this study it was also found that mothers of children with CMA were four times more likely to have a university-level education as compared to mothers of children without allergy [52].

As with all FAs, it is well known that atopic disease in general and particularly atopic dermatitis is an important risk factor for IgE-mediated CMA. This explain why the suspicion of CMA should be stronger in moderate-to-severe atopic dermatitis that starts in the first 6 months of life [53].

Atopic comorbidities such as asthma, especially when inadequately handled, are associated with frequent and severe reactions to milk [54]. Actually, it is still not known whether this is caused by a more severe allergic phenotype or by a barrier function.

Another factor that may have a protective role with respect to food sensitization and allergy later in childhood is increased food diversity in infancy [55]. In fact, Roduit et al. reported that an increased diversity of food within the first year of life might have a protective effect with respect to asthma, FA, and food sensitization [55].

Another risk factor for FA that has been identified is parents’ country of origin. The NHANES study, conducted between 2005 and 2006, compared the risk of food sensitization between US-born children and foreign-born children.

Compared to those born outside the United States, US-born children and adolescents had higher risk of sensitization to any food. Among the foreign-born, those who arrived before 2 years of age had higher odds of food sensitization than those who arrived later [56].

Other potential risk factors that can be examined to reduce/prevent FAs, are: increased hygiene, the influence of the microbiome [57], dietary fat (reduced consumption of omega-3-polyunsaturated fatty acids), reduced consumption of antioxidants, increased use of antacids (reducing digestion of allergens), obesity (being an inflammatory state), and the timing of food introduction in diet (increased risk of delaying oral ingestion of allergens, with environmental exposure, in the absence of oral exposure, leads to sensitization and allergy) [6].

A National Academies of Sciences report (2016) examined many factors and theories suggested to influence the risk of FA [58]. This group examined the “dual allergen exposure hypothesis”, attributed to Gideon Lack, and assessed that there is limited but consistent evidence that an impaired skin barrier plays a role in sensitization as a first step toward FA [6]. This theory suggest that low-dose cutaneous exposure is sensitizing and facilitated by an impaired skin barrier and inflammation. Meanwhile, oral exposure could cause the development of tolerance [6].

Numerous perinatal factors can influence the development of CMA and FAs, but the relationship between them is still controversial [59,60]. Sardecka et al. reported an increased risk of CMA in premature newborns, as previously noted [61], which may result from increased intestinal permeability [52]. The mode of delivery may also influence the development of FAs. The incidence of CMA may be higher in infants born by cesarean section because of the influence on the microbiota and, consequentially, on the immune system. Actually, no relationship between CMA and the type of delivery has ever been observed in studies [52,62].

### 6.1. The Role of Vitamin D

During the last decades, interest in the role of vitamin D in allergic disease has progressively increased. Numerous papers have suggested its possible role as an immune modulator in allergies, especially with respect to lymphocyte activation, antigen receptor function, and signaling pathways [63]. Still, the precise molecular mechanisms involved in vitamin D’s genomic and non-genomic actions remain incompletely defined [64] and not fully understood.

A lack of vitamin D has been associated with an increased risk of FA in many papers [64,65]. Actually, these associations are controversial and need further exploration, as vitamin D sufficiency has also been associated with an increased risk of allergic sensitization.

It is known that populations with lower levels of vitamin D are more susceptible to developing food allergies [66].

An Italian cross-sectional study performed by Lombardi et al. showed that the association between vitamin D levels and allergies was weak, and reported that it was necessary to implement studies involving larger samples to better assess this association [67].

Ecological studies have shown that there is an association between lower sunlight exposure and FA [68].

On the other hand, other papers reported that higher levels of vitamin D could increase the risk of allergic sensitization and FA.

For this reason, the relationship between vitamin D and development of FA is still controversial. Further studies are required to assess the role of vitamin D in the prevention of allergic diseases [68].

### 6.2. The Role of Breastfeeding

It is known that breastfeeding has an important role in the establishment of gut microbiota, nutritional status (with a role in preventing obesity and other nutritional disorders), the immunological system, and neuro-psychomotor development.

Human milk is composed of many molecules with potential immune-modulating roles: antibodies, predominantly secretory immunoglobulin A (s-IgA), cytokines (TGF-β, IL-10, IL-12, thymic stromal lymphopoietin) and chemokines, hormones and growth factors, polyunsaturated fatty acids (PUFAs), glutamine and dietary nucleotides, glycoproteins, oligosaccharides, and microRNA [69,70]. During infancy, it also represents the principal source of protein, fat, calcium, phosphorus, and vitamin B12. For this reason, breastfeeding should not be easily eliminated but encouraged instead [71].

According to the World Health Organization (WHO), breastfeeding is the perfect method of infant feeding; it is recommended exclusively in the first 6 months of life and partially until 2 years of age [72]. Exclusive breastfeeding means that infants should be fed only with breastmilk in the first 6 months of life and receive no other liquid or solids except for vitamins, mineral supplements, or medicines.

The main allergens in CM are casein, α-lactalbumin, and β-lactoglobulin. It is known that casein and α-lactalbumin are natural elements of human milk, while β-lactoglobulin is not present in it [15]. Therefore, the presence of β-lactoglobulin in human milk is caused by maternal ingestion of CM. Actually, it is still not understood whether there is a transfer of proteins such as β-lactoglobulin into breast milk. In any case, it seems that a small fraction of dietary proteins can resist digestion and become eventually allergenic [73].

Current guidelines expressly affirm that in infants with CMA, mothers should be encouraged to continue breastfeeding [69].

As suggested by American Academy of Pediatrics (AAP) and ESPGHAN recommendations, breastfeeding should be continued while solid foods are introduced into the diet [74]. From an allergological standpoint, continuing breastfeeding during solid food introduction and delaying this introduction until at least 17 weeks of age were associated with fewer FAs [75]. Thus, the early introduction of allergenic foods while breastfeeding might be a protective factor against FAs.

As recommended by the AAP, when breastfeeding is not possible or not sufficient, the introduction of CM proteins can be done in the first days or weeks of life through a CM formula [76].

However, the role of early exposure to CM proteins as a risk factor for the development of CMA and FA in general is still not clear. Data regarding a direct relationship between breastfeeding and FA are insufficient [77].

Exclusive breastfeeding for at least 4–6 months was first recommended by the European Section of Pediatrics and the AAP in the early 2000s to prevent FA and CMA in early childhood [78].

In 2008, the AAP affirmed that there was still no convincing evidence that the delayed introduction of allergenic foods beyond 4–6 months had a significant protective effect against the development of allergic disease [78].

A randomized trial study by Saarinen et al. [79] reported that feeding with CM formula in maternity hospitals increased the risk of CMA when compared with feeding with other supplements. However exclusive breastfeeding does not totally eliminate the risk.

Another prospective study on CMA in exclusively breastfed infants by Host et al. [80] underlined that early accidental and occasional exposure to CM proteins may initiate sensitization in predisposed newborns. Subsequent exposure to small amounts of CM proteins in human milk may act as booster doses by eliciting allergic reactions [80].

In a 2018 study, Sardecka et al. [52] found that the risk of CMA in children during the first year of life decreased as a result of a longer period of breastfeeding.

On the contrary, in a prospective large birth cohort study of 13,019 infants, Katz et al. reported that only 0.05% of the newborns who received a regular introduction of CM formula within the first 14 days developed CM-IgE, whereas 1.75% of the infants who started the CM formula between the ages of 105 and 194 days developed CM-IgE [81]. Thus, the authors concluded that early exposure to CM, in addition to breastfeeding, might stimulate tolerance.

Furthermore, a recent case—control study by Onizawa et al. [82] supported the hypothesis that early, regular, and continuous exposure to CM formula, started within the first month of life, can prevent CMA.

Further studies are required in order to confirm the possibility of preventing CMA with regular and early administration of CM formula.

## 7. Natural History of CMA

This paper focuses on CMA as a model of FAs in children.

The rate of resolution of FAs was reviewed by Savage et al. in 2016 [7]; it was found that some allergies have a high rate of resolution in childhood, such as milk (>50% by age 5–10 years), egg (approximately 50% by age 2–9 years), wheat (50% by age 7 years) and soy (45% by age 6 years) allergies, with complete resolution in adolescence. Others that usually persist include peanut (approximately 20% by age 4 years), tree nut (approximately 10%), fish, and shellfish allergies (further studies are necessary).

The natural history of CMA is unique. Resolution is usually common [1] and this contributes to the complication of prevalence estimates.

Actually, there is some heterogeneity in the estimated rate of resolution: studies show different resolution rates (Table 4). Whereas until now there has been a clearly notable heterogeneity among studies, in a combined analysis of patients with CMA in infancy, by the age of 5, 50% developed tolerance and by early adolescence, 75% developed tolerance [1].

A large population cohort study in Israel showed that only 57% of children with CMA resolved their allergy by the age of 4–5, and the majority of these allergies were resolved by age 2 [83].

In contrast, it is important to underline that clinically based studies suggest a poorer prognosis, since they include children who are at higher risk of allergy [17].

Santos et al., in a recent prospective study analyzing Portuguese children with CMA between 1997 and 2006, found that only 41% developed tolerance at the age of 10 [47].

Another important retrospective study of milk allergy by Skripak et al. [84] evidenced that the median age of outgrowing CMA was 10 years. In this case the definition of the development of allergy was having sIgE for milk <3 kUA/L or passing an OFC. Despite important heterogeneity among the studies (CMA diagnosis, study population, clinical features of CMA), it seems that more recent studies have shown less optimistic results, with lower rates of resolution.

It is accepted that 87% of children outgrow CMA by age of 3 [85], but the percentage of patients with persistent allergy has increased, although it was found to vary among publications. This may suggest that the natural history of CMA is changing over time.

Understanding these overall trends and the reasons for the variation has important implications for management and treatment.

**Table 4:** Natural history of CMA in different populations and settings, adapted from Mousan et al. [2].

Authors/Year of Publication	Number of Subjects	Population/Study Design	Tolerance Rate	Age of Tolerance(year)
Høst et al., 2002 [86]	39 (24 IgE-mediated)	General prospective birth cohort	56%	1
			77%	2
			87%	3
			92%	5
			97%	15
Vanto et al., 2004 [44]	162 (95 IgE-mediated)	Referral retrospective	44%	2
			69%	3
			77%	4
Garcia-Ara et al., 2004 [32]	66 IgE-mediated	Referral retrospective	68%	4
Saarinen et al., 2005 [85]	118 (75 IgE-mediated)	General prospective birth cohort	51%	2
			74%	5
			85%	8.6
Skripak et al., 2007 [84]	807 IgE-mediated	Referral retrospective	19%	4
			42%	8
			64%	12
			79%	16
Fiocchi et al., 2008 [87]	112 IgE-mediated	Referral retrospective	52.7%	5
Martorell et al., 2008 [34]	170 IgE-mediated	Referral retrospective	82%	4
Santos et al., 2010 [47]	139 (66 IgE-mediated)	Referral retrospective	41%	2
Ahrens et al., 2012 [88]	52 IgE-mediated	Referral retrospective	61.5%	12
Elizur et al., 2012 [83]	54 IgE-mediated	General prospective birth cohort	57.4%	2
			65%	4
Wood et al., 2013 [89]	293 IgE-mediated	Prospective	53%	5.5
Yavuz et al., 2013 [49]	148 IgE-mediated	Referral retrospective	20%	2
			34%	4
			39%	6
Schoemaker et al., 2015 [21]	55	EuroPrevall, European population-based	57%	2
		Prospective		

## 8. Factors Associated with the Natural History

Many factors have been associated with the natural history of FA with respect to both the development of tolerance and its persistence. Most of these markers have been studied for CMA.

Early identification of patients in which CMA is likely to persist will provide the clinician with useful insights about when and how to start CM reintroduction [22].

As a general rule, non-IgE-mediated allergy resolves more rapidly than IgE-mediated [85].

First of all, it is known that persistent allergy has been connected with more severe clinical features at presentation. Secondly, persistent FA has been linked with an earlier age at diagnosis [83], the presence of other atopic diseases and their severity (allergic rhinitis, asthma, eczema) [49], the presence of other FAs (most commonly egg allergy [49,59]), and a lower threshold dose to trigger a reaction [7]. In addition, reactivity to baked milk (BM) on first exposure is also associated with the persistence of CMA [90].

The natural history of FA varies substantially, and it is known that the variation of food-sIgE or SPT wheal size in time can predict the probability of resolution and the development of tolerance [48]. Generally speaking, larger wheal size on SPT or higher sIgE levels are associated with persistence of FA [89,91]. These associations are consistent with findings from Kim et al. showing that higher sIgE levels at first reaction were the most significant predictors of persistent CMA [92].

Moreover, a strong relation between the food allergen and the persistence of FA has been demonstrated. Some FAs, such as those to milk and egg, have a high likelihood of resolution, whereas sufferers of other allergies such as those to peanut and tree nuts have lower probability of developing tolerance. To sum up, when approaching FA, the clinician should take into account the type of allergen involved, together with SPT results and sIgE levels. Yearly follow-up with repetition of sIgE should be advisable until complete stabilization, as this has a higher prognostic value in predicting tolerance acquisition and the likelihood of passing an OFC [7,93].

### The Role of Baked Milk

The baking process alters protein epitopes which are no longer recognizable by the epitope-specific IgE, leading to decreased allergenicity [1].

Regarding CM, whey proteins such as α-lactalbumin and β-lactoglobulin include conformational epitopes that are heat-labile, whereas casein contains mostly heat-resistant epitopes [94].

As a consequence, BM-driven reactions are indicative of more persistent CMA.

To date, only few papers have compared the development of tolerance to BM as compared to fresh milk, although many retrospective studies have supposed a quicker CMA resolution upon regular ingestion of BM [95].

In a recent study conducted in infants under the age of 2 years, Uncuoglu et al. found that 81% of children with IgE-mediated CMA were baked-milk tolerant [96].

Nowak-Wegrzyn et al., in their study based on 100 children with documented CMA, showed that 75% tolerated BM and were able to include BM products into their diet. After 3 months of BM ingestion, children had significantly smaller SPT wheals as compared to baseline [97].

Kim et al. [92] reported that tolerance to BM products was an advantageous prognostic factor for the development of tolerance to unheated milk. Moreover, the ingestion of BM products seemed to considerably accelerate the development of tolerance as opposed to a strict avoidance diet.

A controlled randomized clinical trial by Esmaeilzadeh et al. further confirmed that regular assumption of BM products accelerated the tolerance to fresh milk [98].

In conclusion, tolerance to BM usually precedes tolerance to fresh milk and represents a reliable predictor for the less severe and persistent CMA phenotype [94].

## 9. Treatment and Oral Immunotherapy

Since the 1930s, scientific literature on CMA has continuously increased. In recent decades, new diagnostic methods and therapies have been developed [99].

From the beginning, the cornerstone of treatment was represented by an elimination diet with strict avoidance of the offending food and its substitution, when possible, with hydrolyzed formulas [100]. However, despite rigid adherence to diet, CM can be inadvertently ingested through processed foods.

In support of this statement, Alonso et al. followed 80 patients with CMA [101] until the achievement of tolerance or up to the age of 18 years (in the case of other allergic diseases), finding accidental ingestion of milk in at least a third of them [102]. This can lead to a feeling of insecurity in children and their parents, significantly worsening their QoL [103].

Given that the avoidance diet as the preferred approach towards CMA has demonstrated limited efficacy, finding a new targeted therapy is pivotal. In response to this need, a novel treatment known as oral immunotherapy (OIT) has emerged through the last decade, changing the history of CMA and FA in general.

Oral tolerance induction testing was first attempted in laboratory animals in 1909 by Besredka, who demonstrated that oral or rectal administration of CM could protect from clinical manifestations such as anaphylaxis [104].

Before this new treatment, patients had to strictly avoid the food allergen, with the consequent reduction in QoL as well as nutritional, social, and psychologic impairment [105].

Nowadays, OIT is the most promising approach for the management of FA [106]. It consists of repeated administration of increasing amounts of the food allergen until reaching a target dose, in order to provide protection against the clinical features and inflammation [107]. Once the target is achieved, the patient must maintain a regular intake of the allergen to preserve the state of desensitization [108]. In fact, “desensitization” refers to a reversible state in which patients can eat higher doses of the food allergen without symptoms as compared with pre-treatment doses [109]. On the other hand, “tolerance” is the ability to introduce the allergen without any adverse reaction once therapy is completed.

Thus, the aim of OIT is to introduce allergenic food into the normal diet, or, in high-risk patients, to prevent life-threatening conditions after inadvertent ingestion [107]. In those patients, OIT is given at a lower dose in order to avoid severe reactions after accidental exposure [107].

OIT is potentially indicated for children with evidence of IgE-mediated CMA and in whom avoidance diet is ineffective, undesirable, or decreases QoL [110]. According to EAACI Guidelines [110], OIT is recommended for persistent CMA for children from around 4 to 5 years of age in order to increase the threshold for clinical manifestations of allergy. Restricting OIT in this age group can be explained by the fact that achievement of tolerance occurs before school age.

OIT has an immunological role in the modulation of humoral and cellular immunity. In particular, humoral changes such as a decrease in IgE levels and a rise in IgG levels, mainly IgG4, have been described. IgG4 could have an antigen-neutralizing effect and decrease basophil and mast cell activation, with the suppression of IgE production [107]. Moreover, OIT drives a reduction in Th2 cell line and Th2 cytokine expression [95].

OIT can be divided into two different phases. The first is the so-called “induction phase” or “dose escalation phase” where the main target is to achieve the tolerated dose [111]. It starts with a small dose, usually in micrograms of allergenic proteins which do not cause a reaction, and continues until the achievement of a target dose or until symptoms preclude further increments [112]. The allergen dose is augmented once or twice a week until reaching a maintenance dose. There are many protocols differing in the amounts of time required: from flash protocols (one week) to slow protocols (>6 months) [107].

Furthermore, the initial dose needs to be adapted to the patient following a personalized medicine approach. For example, low doses should be used in high-risk patients, while a more rapid introduction of the allergenic food could be performed in low-risk patients [113].

The second phase is a “maintenance phase” characterized by repeated intake of maximum tolerated doses of CM [114].

Whether the induced tolerance by OIT is permanent or transient, the long-term effects are still unclear. In agreement with the most recent available studies, not all the children subjected to OIT are able to introduce normal amounts of CM in their diet. Thus, OIT substantially increases the threshold dose necessary to elicit clinical manifestations, resulting in clinical tolerance in a large number of patients [115]. Permanent oral tolerance is reasonably achievable only in a part of the treated patients [116].

Table 5 summarizes the data from various studies on the efficacy of OIT milk.

To date, only few randomized studies have compared the efficacy of immunotherapy to elimination diet, underlining the need for further research with more homogeneous and wider populations.

In this regard, Staden et al. [112] described a population of 47 children with DBPCFC-confirmed CMA, randomly assigned either to OIT or elimination diet. The patients were clinically evaluated at baseline and after a period of 21 months, with repetition of an OFC to assess the persistence of tolerance. At the follow-up OFC, 36% and 35% of the patients in the OIT group and in the control group, respectively, achieved complete tolerance. Although similar results were observed, if we include children who achieved partial tolerance (patients who needed a regular intake of the allergen to maintain tolerance), the efficacy rate of OIT increased to 64% [112]. OIT resulted superior to the elimination diet, with an increased threshold dose for allergic reactions and a reduced burden of severe reactions following accidental ingestion of CM. In a randomized study by Morisset et al. [135], 60 infants with CMA were randomized into an OIT group and a control group treated with elimination diet group. OIT was proposed for those who did not react to the OFC (60 mL of milk). In this study, the rate of spontaneous recovery after 6 months in the control group (60%) resulted considerably lower as compared with that of the OIT group (88.9%). In addition, patients treated with OIT showed lower reactivity in terms of SPT size and lower sIgE levels [135], in accordance with the results of other studies [136].

A recent review with a meta-analysis on OIT in CMA by Martorell Calatayud et al. reported that rates of desensitization after OIT ranged from 36% to 77%, with an estimated tolerance close to 30% [28]. They concluded that this new strategy was an effective and reasonably safe alternative to the avoidance diet. Moreover, their study showed that significantly more patients achieved tolerance with OIT than with the elimination diet [28].

Although many studies highlighted the benefits of OIT, there is still a lack of well-conducted studies concerning the risk of side effects of this novel therapeutic approach.

Since several studies have reported side effect rates of 50–60% [117,136], increasing with exercise and the pollen season, continuous medical supervision during OIT is still mandatory. During OIT mild, localized, and self-limiting side effects usually occur, including oral itching and rhino-conjunctivitis. Symptoms that can lead to the discontinuation of OIT occur in just a small percentage of cases, and include abdominal pain (the most common), wheezing, laryngeal spasms, vomiting, and urticaria [137]. In fact, OIT is usually associated with a modest increase in risks of systemic side effects and a substantial increase in minor local adverse reaction [138]. Among various studies, anaphylactic reactions and the resulting intramuscular administration of epinephrine have been reported in 6.7% to 30.8% of all patients subjected to OIT [28].

The risk–benefit ratio of OIT is still debated. While the efficacy of this approach has been well studied, the evaluation of an effective improvement in the QoL of the patient remains limited to small or uncontrolled studies [139]. In fact, only few studies evaluate children’s perceptions of improved of their QoL as compared to perceptions by parents. A recent paper [139] described how OIT could improve the QoL of (both partially and totally desensitized) food-allergic children and their parents, through the Food Allergy Quality of Life Questionnaire—Child Form (FAQLQ-CF) and the FAQLQ—Parent Form (FAQLQ-PF), respectively. These results were concordant with those of Carraro et al., who gave the same questionnaire to parents of patients with CMA and found a substantial increase in all the investigated areas of QoL (emotional impact, food anxiety, and social and dietary limitations) in children with CMA [140].

Conversely, in 2021 Kauppila et al. reported that the Health-Related Quality of Life (HRQoL) among OIT patients did not differ significantly from that of the age- and gender-standardized general population [141].

In conclusion, OIT is an effective strategy for treating CMA which needs to be performed under the cautious supervision of an experienced specialist. The children who can obtain the greatest benefit from this therapy are those with high sIgE levels and high risk of life-threatening conditions such as anaphylaxis. For these patients, OIT should be considered as a possibility to avoid severe reactions and improve quality of life. However, OIT should be considered as an individualized treatment, and each step needs to be adapted to the specific patient [107].

## 10. Conclusions

CMA represents a model of FAs, since it is the most common and most studied FA in early life. For this reason, clearly understanding its epidemiology, diagnostic criteria, and appropriate treatment can guide the clinician and provide useful insights to better comprehend all other allergies. Ensuring proper diagnosis and prognosis and identifying the possibility of allergy resolution are therefore key components of management.

Thus far there are still no shared tolerance markers for the diagnosis of CMA and FAs in general. Although the negative predictive value of SPT and sIgE is excellent, the OFC, particularly DBPCFC, remains the gold standard for diagnosis. The heterogeneity of diagnostic tools used in literature further limits a reliable estimate of CMA epidemiology and consequentially of its natural history. Appropriately diagnosing CMA is therefore pivotal in understanding its natural history and avoiding unnecessary strict diets that may lead to nutritional deficiencies. In fact, milk is the most important element in children’s diets, providing the necessary intake of fats, proteins, calcium, phosphorus, and vitamin B12. Furthermore, the elimination diet is known to be possibly linked to an increased risk of severe reaction after the inadvertent ingestion of the allergen.

In the last decade there have been many changes in the approach to CMA, which has become proactive. OIT can indeed lead to a change in the natural history of this disease, accelerating tolerance acquisition and the likelihood of passing an OFC.

Therefore, further larger, well-designed, randomized, placebo-controlled trials are necessary to find new diagnosis, prevention, and treatment strategies.

## Figures and Tables

**Table 1:** Main characteristics of CM allergens, adapted from Hochwallner [8].

	Allergen Name	Protein	Concentration (g/L)	Size (kDa)	Prevalence (% of Patients)	Allergenic Activity (% of Patients)
Whey (20%) (5 g/L)	Bos d 4	α–Lactalbumin	1–1.5	14.2	0–67	12
	Bos d 5	β–Lactoglobulin	3–4	18.3	13–62	19
	Bos d 6	Bovine serum albumin	0.1–0.4	66.3	0–76	1
	Bos d 7	Immunoglobulins	0.6–1	160	12–36	
		Lactoferrin	0.09	80	0–35	3
Whole casein (80%) (30 g/L)	Bos d 9	αS1–casein	12–15	23.6	65–100	26
	Bos d 10	αS2–casein	3–4	25.2		
	Bos d 11	β–casein	9–11	24	35–44	35
	Bos d 12	k–casein	3–4	19	35–41	26

**Table 2:** Positive predictive values of 90% and 95% of sIgE levels for a positive challenge.

Author, Year	Age	90%	95%	Method
Sampson and Ho, 1997 [29]	5.2 years	23 kU/L	32 kU/L	CAP system FEIA
Garcia–Ara et al., 2001 [30]	4.8 months	2.5 kU/L	5 kU/L	CAP system FEIA
Sampson, 2001 [31]	3.8 years	15 kU/L	32 kU/L	CAP system FEIA
Garcia–Ara et al., 2004 [32]	13–18 months	1.5 kU/L	2.7 kU/L	CAP system FEIA
	19–24 months	6 kU/L	9 kU/L	
	25–36 months	14 kU/L	24 kU/L	
Celik–Bilgili et al., 2005 [33]	<1 year	25.8 kU/L		CAP system FEIA
Komata et al., 2007 [25]	<1 year		5.8 kU/L	CAP system FEIA
	1 year		38.6 kU/L	
	2 years		57.3 kU/L	
Martorell et al., 2008 [34]	12 months		5.8 kU/L	CAP system FEIA
	18 months		9.8 kU/L	
	24 months		27.5 kU/L	
	36 months		7.4 kU/L	
	48 months		5 kU/L	
Van der Gutgen et al., 2008 [35]	<2.5 years	5 kU/L	7.5 kU/L	CAP system FEIA
Ott. et al., 2008 [36]		52.7 kU/L	66.9 kU/L	CAP system FEIA

**Table 3:** Positive predictive value of 90% and 95% of SPT for a positive challenge.

Author, Year	Age	Ø SPT 90%	Ø SPT 95%	Type of Allergen
Eigenmann and Sampson, 1998 [39]	4.6 years		>5 mm	Glycerinate extract
Sporik et al., 2000 [40]	3 years		>8 mm	Glycerinate extract
	<2 years		>6 mm	
Calvani et al., 2007 [41]	3.6 years		15 mm	Fresh milk
			12 mm	α-Lactalbumin
			8 mm	Casein
			10 mm	β-lactoglobulin
Calvani et al., 2012 [42]	3.7 years		20 mm	Fresh milk
			10 mm	α-Lactalbumin
			7 mm	Casein
			8 mm	β-Lactoglobulin
Onesimo et al., 2013 [38]	2.7 years		4.9 mm	α-Lactalbumin
			4.3 mm	Casein
			5.6 mm	β-Lactaglobulin
Kido et al., 2016 [43]	1.4 years	15 mm		Glycerinate extract

**Table 5:** Efficacy of milk OIT.

Author, Year	Type of Study	Type of Milk	Population (n)	Age (years)	Partial Tolerance	Complete Tolerance
Meglio P. et al., 2004 [117]	Open-label	Fresh CM	21	6–10	14.3% (40–80 mL of CM)	71.4% (200 mL of CM)
Narisety SD. et al., 2009 [118]	Open	Fresh CM	15	6–16		33% (16 g of CM proteins)
Goldberg M. et al., 2015 [119]	Open	Baked CM	14	6.5–12.7		21% (1.3 g of CM proteins)
Takahashi M. et al. 2016 [120]	Open	Microwave heated CM	31	5–17		45.2% (200 mL of CM)
Ebrahimi M. et al.2017 [121]	Open	Fresh CM	14	3.5–7		92.9% (200 to 250 mL of CM)
Skripak et al., 2008 [122]	Randomized, double-blind, placebo-controlled	Fresh CM	13	6–17		30.8% (500 mg of CM proteins)
Longo G. et al., 2008 [123]	Randomized open-label	Fresh CM	30	5–17	54% (5–150 mL of CM)	36% (> 150 mL of CM)
Pajno GB. et al., 2010 [124]	Randomized, placebo controlled	Fresh CM	15	4–10		67% (200 mL ofCM)
Martorell A. et al., 2011 [125]	Randomized, placebo controlled	Fresh CM	30	2–3		90% (200 mL of CM)
Amat F. et al.2017 [126]	Randomized	Baked CMFresh CM	43	3–10	26.8% (0.27–2.5 g of CM proteins)	36.6% (2.72 g of CM proteins)
Maeda M, et al., 2020 [127]	Randomized controlled	Fresh CM	28	3–12		50% (100 mL of CM)
Mota I. et al., 2018 [128]	Prospective	Fresh CM	42	2–18		92% (200 mL of CM)
Berti I. et al., 2019 [129]	Prospective	Fresh CM	73	3–11		97% (150 mL of CM)
De Schryver S. et al., 2019 [130]	Prospective, randomized-controlled	Fresh CM	41	6–18		73.2% (200 mL of CM)
Efron A. et al.,2018 [131]	Retrospective, case-control	Fresh CM	43	1–4		86% (250 mL of CM)
Kauppila T.K. et al., 2019 [132]	Retrospective	Fresh CM	296	5–17		56% (200 mL of CM)
Demir E. et al., 2020 [133]	Retrospective, cohort study	Fresh CM	47	3–13		89.3% (200 mL of CM)
Gruzelle V. et al., 2020 [134]	Retrospective	Baked CM	64	2–16		42.2% (254 mL of CM)
